# A Quality Improvement Approach to Improving Discharge Documentation

**DOI:** 10.1097/pq9.0000000000000428

**Published:** 2022-01-26

**Authors:** Sumeet L. Banker, Divya Lakhaney, Benjamin S. Hooe, Teresa A. McCann, Connie Kostacos, Mariellen Lane

**Affiliations:** From the * Division of Critical Care and Hospital Medicine, Department of Pediatrics, Columbia University Irving Medical Center, New York, N.Y.; † Division of Child and Adolescent Health, Department of Pediatrics, Columbia University Irving Medical Center, New York, N.Y.

## Abstract

**Introduction:**

Accurate discharge documentation is critical to ensuring a safe and effective transition of care following hospitalization, yet many discharge summaries do not meet consensus standards for content. A local needs assessment demonstrated gaps in documentation of 3 essential elements: discharge diagnosis, discharge medications, and follow-up appointments. This study aimed to increase the completion of three discharge elements from a baseline of 45% by 20 percentage points over 16 months for patients discharged from the general pediatrics service.

**Methods:**

Ten discharge summaries were randomly selected and analyzed during each successive 2-week time period. Plan-Do-Study-Act cycles aimed to improve provider knowledge of essential discharge summary content, clarify communication during rounds, and create electronic health record shortcuts and quick-reference tools.

**Results:**

The percentage of discharge summaries containing all 3 required elements increased from 45% to 73%. Specifically, documentation increased for discharge diagnosis (65%–87%), discharge medications (71%–90%), and follow-up appointments (88%–93%). There was no significant delay in discharge summary completion.

**Conclusions:**

Discharge summaries are meaningfully and sustainably improved through provider education, workflows for clear communication, and electronic health record optimization.

## Abstract 2

Supplemental Digital Content is available in the text.

## INTRODUCTION

Accurate discharge documentation is critical to ensuring a safe and effective transition of care from hospital to home. Lapses in discharge-related communication have led to decreased patient safety, lower clinician satisfaction, higher resource utilization, and preventable adverse events after discharge.^1–3^ A high-quality discharge summary is an established pediatric transition of care quality measure that is an essential component of a hospital discharge bundle and ultimately contributes toward a safer discharge.^4–6^ Professional organizations such as the Joint Commission and the American Academy of Pediatrics have established discharge summary standards.^7,8^ Additionally, the Accreditation Council for Graduate Medical Education and the American Board of Pediatrics state in their milestones report that all residents should be able to “provide transfer of care that ensures seamless transitions.”^9^

Despite these criteria, there is a wide variation in discharge summary content, and many do not meet these basic standards.^10–15^ A local needs assessment demonstrated deficiencies in the inclusion of three required elements: discharge diagnosis, discharge medications, and follow-up appointments.^10^ Deficits in these elements have been shown to lead to poor patient outcomes and increased healthcare costs. Lack of diagnosis clarity contributes to low health literacy, leading to higher emergency room use.^16^ Medication inaccuracies lead to patient delay in obtaining medications and possible patient discomfort and/or clinical deterioration^17^ and adverse drug reactions.^18,19^ Additionally, scheduling follow-up appointments before discharge leads to increased primary care follow-up and potentially decreased readmission rate.^20^

Previous studies have demonstrated improvement in the completion and quality of discharge summaries following educational interventions targeted toward medical trainees,^14,15,21–23^ and some have utilized quality improvement (QI) methodology.^6,24,25^ However, few studies have demonstrated a sustainable change in pediatric settings in the era of electronic health record (EHR) use.^6^ This QI study aimed to increase the completion of 3 required hospital discharge summary elements (discharge diagnosis, discharge medications, and follow-up appointments) from a baseline of 45% by 20 percentage points over 16 months for patients discharged from the general pediatrics service.

## METHODS

### Study Design

This QI project was performed from June 2018 through September 2019 at an urban academic tertiary children’s hospital with 260 inpatient beds and roughly 2,400 annual discharges from the general pediatrics inpatient service. The intervention period was 12 months, followed by 3 months of data collection to assess sustainability. We analyzed the EHR-based discharge summaries of patients discharged from the general pediatrics inpatient service. The inpatient service comprises 2 independent teaching services staffed by attending physicians, house staff, and medical students. The first-year house staff complete 4-week rotations on this service and come from various training programs including pediatrics, pediatric neurology, anesthesiology, and family medicine. The second and third-year house staff from the categorical pediatrics program function in a supervisory role. Over the study period, attending physicians completed 2-week blocks of service. They were either faculty with a primary appointment in pediatric hospital medicine or faculty with a primary pediatric ambulatory medicine appointment.

### Standard Processes

House staff complete a templated hospital discharge summary in the EHR (Allscripts, Chicago, Ill.) for every patient upon discharge. Discharge summaries include vital elements required by the Joint Commission^7^ and recommended by the American Academy of Pediatrics Value in Pediatrics Transition of Care Collaborative.^8^ The template has discrete fields for several elements (eg, discharge date) and select open-ended free-text sections (eg, hospital course). Though the template has fields for specific essential elements, they are not required for completion—fields may be left blank, or elements may be found in the incorrect section. The admission date is automatically populated, and the remaining fields must be entered manually. House staff of all levels contribute toward electronic discharge summaries throughout a patient’s hospitalization. A resident ultimately signs the summary, typically on the day of discharge, and routes it to the attending physician of record, who co-signs the note and has an opportunity to edit content for accuracy and clarity.

### Improvement Team

**Fig. 1.**
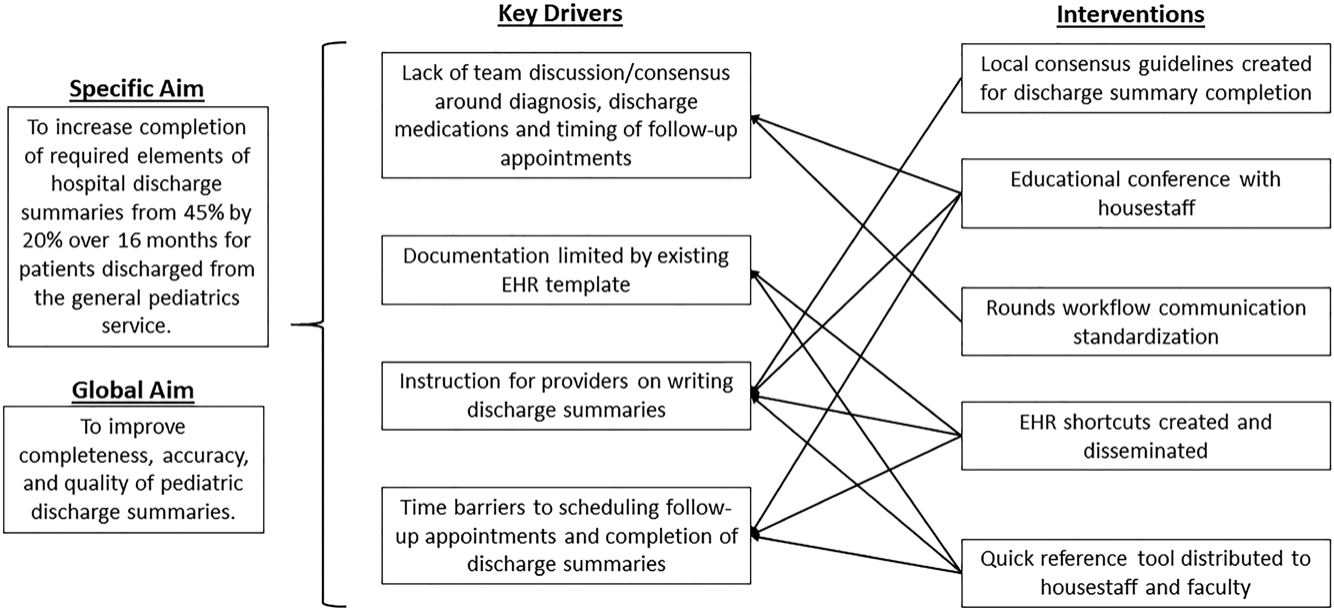
Key drivers in discharge summary completion.

Study investigators formed a diverse team of physician stakeholders from various groups: pediatric hospital medicine faculty, ambulatory medicine faculty, pediatric house staff, and chief pediatric residents. After an extensive literature review, stakeholder meetings with nursing and pharmacy staff, and team members’ expertise, investigators identified key drivers of discharge summary completion (Fig. 1). The study team met regularly to discuss possible Plan-Do-Study-Act (PDSA) cycles and review qualitative feedback and quantitative data from tests of change. PDSA cycles included: improving provider knowledge of essential elements of pediatric discharge summaries,^8^ improving consensus during rounds and clinical care, creating shortcuts within the EHR, and distributing quick-reference tip sheets to providers.

### Interventions

#### Local Consensus

An initial stakeholder meeting was held between hospital medicine faculty, ambulatory medicine faculty, chief residents, and house staff to review the discharge summary template, the step-by-step process for completion, and develop a standardized rubric regarding summary completion. This standardized rubric was shared with the house staff and faculty and used to develop provider materials to establish clear expectations.

#### House staff Education

Investigators developed a hands-on educational session for house staff, which included: (1) a didactic portion on the history and importance of the transition of care communication/documentation; (2) presentation of local data obtained via a needs assessment^10^; and (3) an interactive small-group workshop where trainees evaluated de-identified discharge summaries based on the standardized rubric and discussed specific feedback to improve quality. This session was delivered by three faculty members and one senior resident during a 1-hour protected conference time slot at the start of each training year. First-year house staff were the targets of this training, though some senior pediatrics house staff also attended to reinforce their skills.

#### Rounds Workflow Standardization

We developed rounding cards to increase consensus between attending physicians, house staff, and families (**Figure 1, Supplemental Digital Content 1,** which displays rounds communication cards (a) front and (b) back, http://links.lww.com/PQ9/A273). These cards prompted senior team members to explicitly discuss and agree upon essential discharge care elements, including diagnosis, plan of care (including follow-up appointments), anticipated discharge medications and equipment, and discharge date. The cards were completed by the first-year house staff and given to families during rounds to reduce uncertainty among families and house staff, thus improving consensus and documentation of these elements. Rounding cards were reviewed with families daily because stakeholder meetings revealed that discharge summaries are often initiated before the day of discharge.

#### EHR Shortcuts

We developed acronym expanders (or “dot-phrases”) to standardize discharge care plans for specific common diagnoses (eg, bronchiolitis, asthma exacerbation, and gastroenteritis). These dot-phrases were shared with all house staff via the institution’s EHR. Given that the discharge summary template is common to adult and pediatric discharges and an upcoming transition to a new EHR, structural modifications to the template were not possible during the study period.

### Reference Tool for Providers

**Fig. 2.**
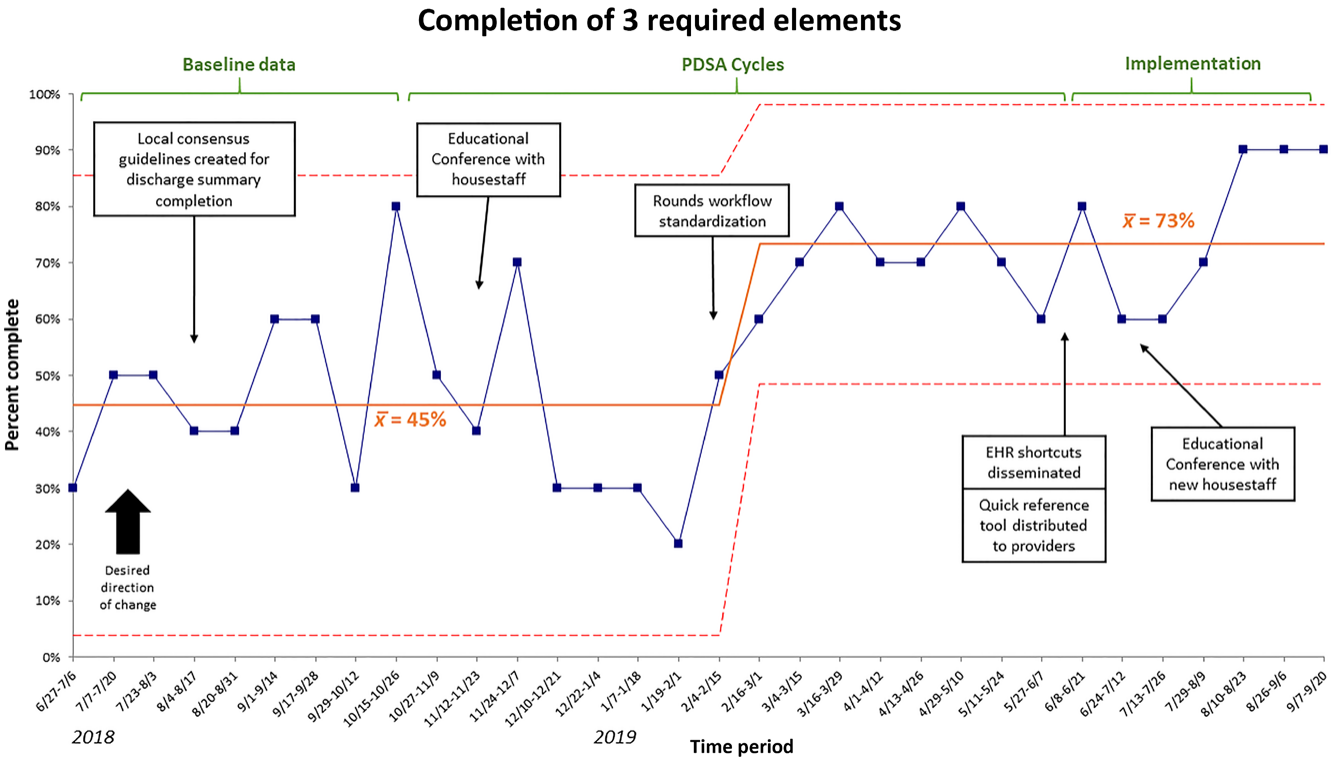
Statistical process control chart (p-chart) of completion rate of all 3 required elements (primary outcome).

A laminated pocket-guide was developed and distributed to house staff and attending physicians. This reference tool guided providers along each section of the discharge summary and included useful EHR shortcuts and acronym expanders to increase efficiency and standardization of language used. The guides were given to individual providers and posted in common house staff work areas (**Figure 2, Supplemental Digital Content 2,** which displays reference tool for providers, http://links.lww.com/PQ9/A274).

### Measures and Data Collection

The primary outcome measure was the percentage of selected discharge summaries in a 2-week period that contained all three required elements: discharge diagnosis, discharge medications, and follow-up appointments. The investigators chose these three elements after baseline data review^10^ revealed the greatest opportunity to improve these essential elements of discharge summaries within the current EHR template.^8^ As a balancing measure, investigators recorded delays in discharge summary completion: the number of discharge summaries that house staff completed after the date of discharge. We also tracked the total number of house staff authors per discharge summary as a contextual indication of changes in house staff workload and handoffs.

All data collection was by manual chart review. The investigators randomly selected ten discharge summaries from a list of all patients discharged from the general pediatrics inpatient service during the previous 2-week period. Investigators analyzed discharge summaries for three selected elements and their date of completion. Elements were noted as completed or omitted based on whether the information was within the appropriate designated field (ie, the diagnosis was noted as omitted if found in the hospital course but not in the “discharge diagnosis” field).

### Analysis

The p-chart, a type of statistical process control chart, was used to assess the impact of interventions, with the following criteria used to determine positive special cause variation due to changes in the process: >7 values in a row above the baseline mean or ≥6 steadily increasing values in a row.^26^ This study was approved by the Columbia University Institutional Review Board.

## RESULTS

There were 320 individual discharge summaries evaluated over 32 two-week periods during the 64-week study period.

### Outcome Measures

**Fig. 3.**
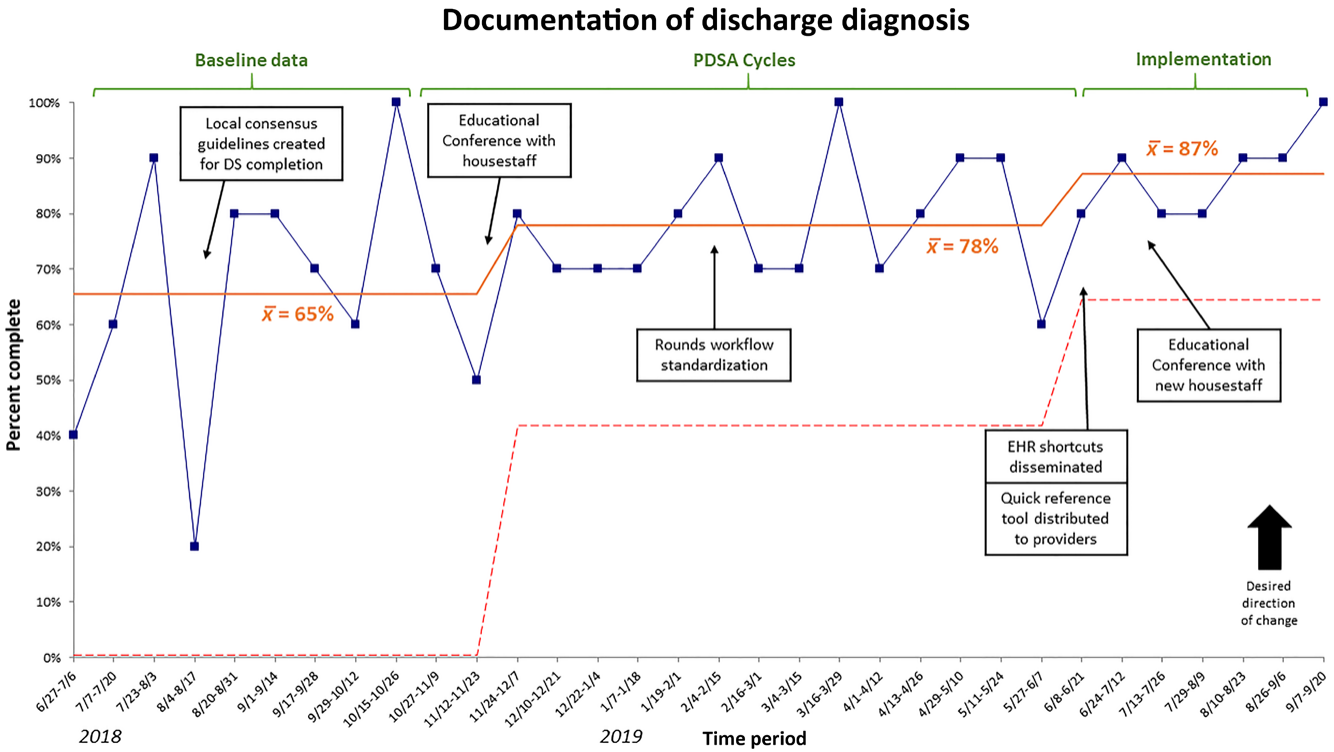
Statistical process control chart (p-chart) of documentation of discharge diagnosis.

The proportion of discharge summaries containing all three required elements increased from 45% to 73% over the study period (Fig. 2). This change met the statistical threshold for special cause variation based on >7 consecutive periods above the baseline mean. Among individual elements, discharge diagnosis documentation increased from 65% to 87% (Fig. 3), discharge medications increased from 71% to 90%, and follow-up appointments increased from 88% to 93%.

Early interventions such as establishing consensus among stakeholders and sharing expectations with house staff via education were necessary to establish a foundation. However, standardizing rounds-based communication appeared to be most impactful because the sustained improvement over baseline occurred after this intervention.

### Balancing Measures

Of the 320 discharge summaries sampled in the study period, only one was completed after the date of discharge. Therefore, there was no delay in discharge summary completion by house staff as a result of the interventions. On average, there were 2.2 house staff authors per discharge summary over the entire study period compared to a baseline of 2.1 authors; there were no major shifts in the average number of house staff authors over the study period, nor were there any significant changes to the house staff schedule that may have affected workload or handoffs among providers.

## DISCUSSION

This QI project achieved our aim of an absolute increase of over 20% in the inclusion of three essential elements within the discharge summary without increasing the time demand on house staff. We achieved improvement over 13 months and implementation sustained through the transition to new house staff, thereby moderating the effect of new learners between academic years. This study adds to the scant literature of using QI methodology to achieve sustained improvement in discharge summaries. Our discharge summary completion rate is similar to other studies in this area, which report a completion rate between 70% and 80%.^6^ Our PDSA cycle innovations were straightforward and inexpensive and may be scaled to different practice settings regardless of hospital size, clinical volume, or patient population.

The interventions were successful at the level of providers as well as at the system level. Educational interventions have been well-studied and shown to improve discharge summaries’ quality and content at the provider level.^14,15,21,22^ House staff and attending physicians’ perceptions of note quality are discordant across many domains.^27^ Thus, engaging both faculty and house staff in establishing a standardized consensus rubric for discharge summaries and providing feedback were essential steps in this study. To maximize scalability and decrease time burden on faculty providing feedback to house staff, the study team utilized a group feedback approach with house staff,^15^ However, individual feedback has also been shown in other studies to improve discharge summary quality.^21,22,24^ Our improvement sustained past the training year transition in July, which is often a challenge in teaching institutions where new providers are oriented each year to the institution’s policies and procedures.

Of the innovations, creating a standard rounds workflow for communication of essential elements seemed to have a meaningful impact based on sustained improvement in the primary outcome (Fig. 2). Implementing a standardized process to explicitly achieve verbal agreement amongst the medical team regarding the care plan’s critical elements may have led to an improved consensus among house staff and better completion of those elements within discharge summaries. There was notable enthusiasm from our house staff around this intervention for increasing consensus with the attending and providing the family with a tangible summary of the care plan. Previous studies show that information-sharing provides team members with an explicit shared mental model and improves clinical outcomes.^28,29^ House staff and attending physicians may not be on the same page about details of care given studies have shown 42% concordance in orthopedic procedural codes^30^ and under 70% agreement in primary diagnosis and discharge medications, even after rounds on a pediatrics inpatient service (G. Ru, MD and S.L. Banker, MD, MPH, unpublished data, May 2020). Simple structures may assure communication quality, such as closed-loop communication, structured information transmission (eg, SBAR), or structured handoffs.^31,32^

The widespread adoption of EHR systems presents an opportunity to incorporate system-level enhancements within templated discharge summaries, which were not possible in earlier studies where discharge summaries were dictated.^11,15,21,24^ Still, templated documents are not perfect, and discharge summaries may be signed with fields left blank or with information in the wrong places despite agency standards. Continued optimization and hard-stop requirements may help to mitigate these errors. Other studies have shown the potential of pediatric EHR-based templates and dot-phrases to improve written provider handoffs,^6,33^ documentation and timing of neurovascular exams,^34^ documentation of gun safety discussion during well child care,^35^ documentation of patient-focused teratogen education,^36^ and documentation of inpatient disposition recommendations related to children admitted with diabetic ketoacidosis.^37^ Provider-focused EHR education can lead to improved quality, readability, and accuracy of documentation, as well as fewer medical errors.^38^

Future studies should assess the successful dissemination of the completed discharge summaries to the primary care provider and best practices for the verbal transition of care communication with primary care providers to complement the written summary. Additionally, as communication deficits around transitions of care have led to medical errors, studies should assess whether improvements in discharge summaries lead to decreased medical errors. Researchers may also apply these interventions to improve discharge summaries in other fields of medicine. Additional work could re-examine the effect of clinical workload on the quality of discharge summaries in the current landscape of duty hours restrictions and near-universal EHR use,^39^ and continue to improve house staff efficiency around documentation.^40^

There are limitations to this QI project. First, this project was implemented within a single academic medical center with a specific EHR, restricting the findings’ generalizability. However, the interventions were relatively simple and reproducible in different institutions and across other EHRs. Though we achieved consistency across the training years, monitoring for longer-term sustainability may also be of benefit. Second, we did not evaluate the discharge summary’s accuracy, as was done in previous studies by the authors. Third, as the authors did not collect data regarding the complexity of hospitalizations, it is possible that patients and hospitalizations in the postintervention period were less complex and led to an improvement in outcomes. However, the randomization scheme sought to limit this selection bias, and hospital-level data does not support a shift in patient complexity over this study period. Fourth, our improvement team did not include representatives from nursing or pharmacy. However, meetings were held with these stakeholders, and physicians remain the sole authors of discharge summaries. Last, other institutional efforts outside of our study interventions may have led to improvements in discharge summaries. For example, in July 2019, our hospital hired a social work assistant to help schedule hospital follow-up appointments. However, this likely had a small effect on our outcome, given that this element’s documentation remained stable throughout the study period.

## CONCLUSIONS

Discharge documentation represents a critical communication tool in the transition of care, often overlooked in the haste of discharge. Despite a relatively inflexible EHR template, we meaningfully and sustainably improved the completion of this document past the start of a new academic year using QI methodology through provider education, standardization of rounds-based communication, and EHR shortcut creation. Future research should include obtaining feedback from primary care providers regarding the improved discharge summaries and tracking reductions in medical errors resulting from improved transitions of care.

## DISCLOSURE

The authors have no financial interest to declare in relation to the content of this article.

## ACKNOWLEDGMENTS

The authors would like to acknowledge Dr. Melissa Stockwell for her assistance with this study’s design.

## Supplementary Material



## References

[1] Kripalani S LeFevre F Phillips CO. Deficits in communication and information transfer between hospital-based and primary care physicians: implications for patient safety and continuity of care. JAMA. 2007;297:831–841. 17327525 10.1001/jama.297.8.831

[2] Moore C Wisnivesky J Williams S. Medical errors related to discontinuity of care from an inpatient to an outpatient setting. J Gen Intern Med. 2003;18:646–651. 12911647 10.1046/j.1525-1497.2003.20722.x PMC1494907

[3] Forster AJ Murff HJ Peterson JF. The incidence and severity of adverse events affecting patients after discharge from the hospital. Ann Intern Med. 2003;138:161–167. 12558354 10.7326/0003-4819-138-3-200302040-00007

[4] Leyenaar JK Bergert L Mallory LA. Pediatric primary care providers’ perspectives regarding hospital discharge communication: a mixed methods analysis. Acad Pediatr. 2015;15:61–68. 25444655 10.1016/j.acap.2014.07.004 PMC4371737

[5] Leyenaar JK Desai AD Burkhart Q. Quality measures to assess care transitions for hospitalized children. Pediatrics. 2016;138:e20160906. 27471218 10.1542/peds.2016-0906 PMC9534577

[6] Mallory LA Osorio SN Prato BS. Project IMPACT pilot report: feasibility of implementing a hospital-to-home transition bundle. Pediatrics. 2017;139:e20154626. 28202769 10.1542/peds.2015-4626

[7] Joint Commission on Accreditation of Healthcare Organizations. Hospital Accreditation Standards. Standard IM 6.10 EP 7-9. The Joint Commission; 2008. 10538769

[8] Coghlin DT Leyenaar JK Shen M. Pediatric discharge content: a multisite assessment of physician preferences and experiences. Hosp Pediatr. 2014;4:9–15. 24435595 10.1542/hpeds.2013-0022 PMC4128020

[9] The Accreditation Council for Graduate Medical Education and the American Board of Pediatrics. The Pediatric Milestones Project. 2017. Available at: https://www.acgme.org/Portals/0/PDFs/Milestones/PediatricsMilestones.pdf. Accessed July 22, 2020.

[10] Lakhaney D Banker SL. An evaluation of the content of pediatric discharge summaries. Hosp Pediatr. 2020;10:949–954. 33097565 10.1542/hpeds.2020-0148

[11] Legault K Ostro J Khalid Z. Quality of discharge summaries prepared by first year internal medicine residents. BMC Med Educ. 2012;12:77. 22894637 10.1186/1472-6920-12-77 PMC3532338

[12] Tsopra R Wyatt JC Beirne P. Level of accuracy of diagnoses recorded in discharge summaries: a cohort study in three respiratory wards. J Eval Clin Pract. 2019;25:36–43. 30105889 10.1111/jep.13020

[13] Sarzynski E Hashmi H Subramanian J. Opportunities to improve clinical summaries for patients at hospital discharge. BMJ Qual Saf. 2017;26:372–380. 10.1136/bmjqs-2015-005201 27154878

[14] Ming D Zietlow K Song Y. Discharge summary training curriculum: a novel approach to training medical students how to write effective discharge summaries. Clin Teach. 2019;16:507–512. 30378265 10.1111/tct.12960

[15] Talwalkar JS Ouellette JR Alston S. A structured workshop to improve the quality of resident discharge summaries. J Grad Med Educ. 2012;4:87–91. 23451314 10.4300/JGME-D-10-00249.1 PMC3312541

[16] Morrison AK Schapira MM Gorelick MH. Low caregiver health literacy is associated with higher pediatric emergency department use and nonurgent visits. Acad Pediatr. 2014;14:309–314. 24767784 10.1016/j.acap.2014.01.004 PMC4003496

[17] Wong JD Bajcar JM Wong GG. Medication reconciliation at hospital discharge: evaluating discrepancies. Ann Pharmacother. 2008;42:1373–1379. 18780806 10.1345/aph.1L190

[18] Forster AJ Murff HJ Peterson JF. Adverse drug events occurring following hospital discharge. J Gen Intern Med. 2005;20:317–323. 15857487 10.1111/j.1525-1497.2005.30390.x PMC1490089

[19] Bates DW Boyle DL Vander Vliet MB. Relationship between medication errors and adverse drug events. J Gen Intern Med. 1995;10:199–205. 7790981 10.1007/BF02600255

[20] Marcondes FO Punjabi P Doctoroff L. Does scheduling a postdischarge visit with a primary care physician increase rates of follow-up and decrease readmissions? J Hosp Med. 2019;14:E37–E42. 31532749 10.12788/jhm.3309

[21] Myers JS Jaipaul CK Kogan JR. Are discharge summaries teachable? The effects of a discharge summary curriculum on the quality of discharge summaries in an internal medicine residency program. Acad Med. 2006;81(10 suppl):S5–S8. 10.1097/01.ACM.0000236516.63055.8b 17001135

[22] Key-Solle M Paulk E Bradford K. Improving the quality of discharge communication with an educational intervention. Pediatrics. 2010;126:734–739. 20876170 10.1542/peds.2010-0884

[23] Black M Colford CM. Transitions of care: improving the quality of discharge summaries completed by internal medicine residents. MedEdPORTAL. 2017;13:10613. 30800815 10.15766/mep_2374-8265.10613 PMC6338163

[24] Axon RN Penney FT Kyle TR. A hospital discharge summary quality improvement program featuring individual and team-based feedback and academic detailing. Am J Med Sci. 2014;347:472–477. 24845304 10.1097/MAJ.0000000000000171

[25] Bischoff K Goel A Hollander H. The housestaff incentive program: improving the timeliness and quality of discharge summaries by engaging residents in quality improvement. BMJ Qual Saf. 2013;22:768–774. 10.1136/bmjqs-2012-001671 23704085

[26] Benneyan JC Lloyd RC Plsek PE. Statistical process control as a tool for research and healthcare improvement. Qual Saf Health Care. 2003;12:458–464. 14645763 10.1136/qhc.12.6.458 PMC1758030

[27] Stewart E Kahn D Lee E. Internal medicine progress note writing attitudes and practices in an electronic health record. J Hosp Med. 2015;10:525–529. 26138806 10.1002/jhm.2379

[28] Page JS Lederman L Kelly J. Teams and teamwork in cancer care delivery: shared mental models to improve planning for discharge and coordination of follow-up care. J Oncol Pract. 2016;12:1053–1058. 27858547 10.1200/JOP.2016.013888

[29] Westli HK Johnsen BH Eid J. Teamwork skills, shared mental models, and performance in simulated trauma teams: an independent group design. Scand J Trauma Resusc Emerg Med. 2010;18:47. 20807420 10.1186/1757-7241-18-47 PMC2939527

[30] Murphy RF Littleton TW Throckmorton TW. Discordance in current procedural terminology coding for foot and ankle procedures between residents and attending surgeons. J Surg Educ. 2014;71:182–185. 24602706 10.1016/j.jsurg.2013.07.005

[31] Weller J Boyd M Cumin D. Teams, tribes and patient safety: overcoming barriers to effective teamwork in healthcare. Postgrad Med J. 2014;90:149–154. 24398594 10.1136/postgradmedj-2012-131168

[32] Haig KM Sutton S Whittington J. SBAR: a shared mental model for improving communication between clinicians. Jt Comm J Qual Patient Saf. 2006;32:167–175. 16617948 10.1016/s1553-7250(06)32022-3

[33] Koo JK Moyer L Castello MA. Improving accuracy of handoff by implementing an electronic health record-generated tool: an improvement project in an academic neonatal intensive care unit. Pediatr Qual Saf. 2020;5:e329. 32766500 10.1097/pq9.0000000000000329 PMC7360222

[34] Cao J Farmer R Carry PM. Standardized note templates improve electronic medical record documentation of neurovascular examinations for pediatric supracondylar humeral fractures. JB JS Open Access. 2017;2:e0027. 30229228 10.2106/JBJS.OA.17.00027 PMC6133146

[35] Gastineau KA Stegall CL Lowrey LK. Improving the frequency and documentation of gun safety counseling in a resident primary care clinic. Acad Pediatr. 2021;21:117–123. 32673765 10.1016/j.acap.2020.07.013

[36] Cooper AM Horwitz M Becker ML. Improving the safety of teratogen prescribing practices in a pediatric rheumatology clinic. Pediatrics. 2019;143:e20180803. 30837294 10.1542/peds.2018-0803

[37] Kaushal T Lord K Olsen R. Improving disposition decision-making for pediatric diabetic ketoacidosis: a quality improvement study. Pediatr Qual Saf. 2020;5:e260. 32426627 10.1097/pq9.0000000000000260 PMC7190258

[38] Robinson KE Kersey JA. Novel electronic health record (EHR) education intervention in large healthcare organization improves quality, efficiency, time, and impact on burnout. Medicine (Baltimore). 2018;97:e12319. 30235684 10.1097/MD.0000000000012319 PMC6160120

[39] Coit MH Katz JT McMahon GT. The effect of workload reduction on the quality of residents’ discharge summaries. J Gen Intern Med. 2011;26:28–32. 20697967 10.1007/s11606-010-1465-z PMC3024111

[40] Oxentenko AS West CP Popkave C. Time spent on clinical documentation: a survey of internal medicine residents and program directors. Arch Intern Med. 2010;170:377–380. 20177042 10.1001/archinternmed.2009.534

