# Supplementary material for: A Quality Improvement Approach to Improving Discharge Documentation
Source: Pediatr Qual Saf. 2022 Jan 26;7(1):e428. doi: 10.1097/pq9.0000000000000428 (PMC10997293; doi:10.1097/pq9.0000000000000428)
Supplement: Supplementary file 1 [file pqs-7-e428-s001.pdf]

Today's date: ___________

Your child was admitted to the hospital with the following problem/diagnosis: ______________________________

Your child's plan of care for today is:

1. ____
2. ____
3. ____

We estimate that your child will be discharged:

today   tomorrow   in 2-3 days   over 3 days from now

[See back for list of medications]

Current medications:

Other notes:
