# Supplementary material for: A Quality Improvement Approach to Improving Discharge Documentation
Source: Pediatr Qual Saf. 2022 Jan 26;7(1):e428. doi: 10.1097/pq9.0000000000000428 (PMC10997293; doi:10.1097/pq9.0000000000000428)
Supplement: Supplementary file 2 [file pqs-7-e428-s002.pdf]

How to Complete a Discharge Summary

Key Elements	Description
**Admission & Discharge Dates**	Discharge date should be updated on discharge
**Discharge Diagnosis**	Should reflect the underlying diagnosis (if known) that prompted admission, not signs or symptoms (i.e. "gastroenteritis" not diarrhea; "bronchiolitis" not respiratory distress)
**Follow-up Appointments**	Each should include:<br>• **Name** of provider or clinic<br>• Follow-up **date and time** OR **timeframe**<br>• Address and/or phone number of clinic
**Brief Hospital Course**	1. Summary statement/discharge assessment<br>2. Hospital course **by problem**<br>• **Brief** discussion of the evaluation, treatment, and outcome of each problem<br>• **Clinical reasoning** for the decisions made, particularly if there was ambiguity<br>3. Physical exam at the time of discharge<br>*Acronym Expander **.dcsummary***
**Immunizations Given**	List immunizations administered during hospitalization
**Instructions for Provider**	1. Follow-up plans for pending labs<br>2. Further coordination of care<br>• Specialist appointments that need to be scheduled<br>• Labs/imaging studies to be performed as an outpatient (i.e. renal ultrasound, repeat CBC)<br>3. Additional notes pertinent for a PMD follow-up visit<br>*Acronym Expander **.dcprovider***
**Instructions for Patient/ Family/Caregiver**	1. Reason for admission<br>2. Pertinent changes<br>3. Additional pertinent home care or wound care instructions<br>*Acronym Expander **.dcpatient** & for common diagnoses (.dcasthma, .dcgastro, .dcbronchiolitis, .dcpneumonia)**
**Pending Labs**	List any pertinent pending labs the PMD should follow
**Discharge Medications ("Med Rec")**	Med Rec must be completed prior to completion of the discharge summary in order to pull in discharge meds

Import Acronym Expanders from Benjamin Hooe
